# The Effect of Whey Protein Supplementation on Myofibrillar Protein Synthesis and Performance Recovery in Resistance-Trained Men

**DOI:** 10.3390/nu12030845

**Published:** 2020-03-21

**Authors:** Robert W. Davies, Joseph J. Bass, Brian P. Carson, Catherine Norton, Marta Kozior, Daniel J. Wilkinson, Matthew S. Brook, Philip J. Atherton, Ken Smith, Philip M. Jakeman

**Affiliations:** 1 Department of Physical Education & Sport Sciences, University of Limerick, V94 T9PX Limerick, Ireland; joseph.bass2@nottingham.ac.uk (J.J.B.); brian.carson@ul.ie (B.P.C.); catherine.norton@ul.ie (C.N.); marta.kozior@ul.ie (M.K.); phil.jakeman@ul.ie (P.M.J.); 2 Food for Health Ireland (FHI), Centre for Interventions in Infection, Inflammation & Immunity, University of Limerick, V94 T9PX Limerick, Ireland; 3 Medical Research Council (MRC) and Arthritis Research United Kingdom (ARUK) Centre for Musculoskeletal Aging Research and National Institute for Health Research, Nottingham Biomedical Research Centre, University of Nottingham, Nottingham NG7 2UH, UK; daniel.wilkinson@nottingham.ac.uk (D.J.W.); matthew.brook@nottingham.ac.uk (M.S.B.); philip.atherton@nottingham.ac.uk (P.J.A.); ken.smith@nottingham.ac.uk (K.S.); 4 Health Research Institute (HRI), University of Limerick, V94 T9PX Ireland, Ireland

**Keywords:** dietary protein, deuterium oxide, exercise performance, humans, skeletal muscle, whey protein.

## Abstract

Background: The aim of this study was to investigate the effect of whey protein supplementation on myofibrillar protein synthesis (myoPS) and muscle recovery over a 7-d period of intensified resistance training (RT). Methods: In a double-blind randomised parallel group design, 16 resistance-trained men aged 18 to 35 years completed a 7-d RT protocol, consisting of three lower-body RT sessions on non-consecutive days. Participants consumed a controlled diet (146 kJ·kg^−1^·d^−1^, 1.7 g·kg^−1^·d^−1^ protein) with either a whey protein supplement or an isonitrogenous control (0.33 g·kg^−1^·d^−1^ protein). To measure myoPS, 400 ml of deuterium oxide (D_2_O) (70 atom %) was ingested the day prior to starting the study and m. vastus lateralis biopsies were taken before and after RT-intervention. Myofibrillar fractional synthetic rate (myoFSR) was calculated via deuterium labelling of myofibrillar-bound alanine, measured by gas chromatography-pyrolysis-isotope ratio mass spectrometry (GC-Pyr-IRMS). Muscle recovery parameters (i.e., countermovement jump height, isometric-squat force, muscle soreness and serum creatine kinase) were assessed daily. Results: MyoFSR PRE was 1.6 (0.2) %∙d^−1^ (mean (SD)). Whey protein supplementation had no effect on myoFSR (*p* = 0.771) or any recovery parameter (*p* = 0.390–0.989). Conclusions: Over an intense 7-d RT protocol, 0.33 g·kg^−1^·d^−1^ of supplemental whey protein does not enhance day-to-day measures of myoPS or postexercise recovery in resistance-trained men.

## 1. Introduction

Over recent decades, the role that nutrition and/or exercise has on muscle protein synthesis (MPS) has been guided by the use of isotopically labelled amino acid tracers in acute lab-based studies. Thus, it is known that a single bout of resistance-exercise training (RT) stimulates a postexercise increase in mixed-muscle and myofibrillar protein synthesis (myoPS) [1,2,3]. When high-quality dietary protein (i.e., rapidly digested and absorbed protein, rich in essential amino acids (EAA)) is ingested, in temporal proximity to RT, a further synergistic increase in myoPS occurs, which can be sustained for 4 to 5 h postingestion [4,5,6,7]. Following exercise, this acute increase in myoPS is thought to facilitate muscle reconditioning (i.e., repair and remodeling) and net protein accretion, which is important for individuals who engage in prolonged periods of intense RT. However, the extent to which acute lab-based measures of myoPS can predict long-term physical changes in muscle has been challenged [8].

Recent technical advances in mass spectrometry have led to the (re)introduction of the deuterium-oxide (D_2_O) labelling methods to measure muscle protein fractional synthetic rate (FSR) [9]. As D_2_O can be orally administered with a relatively slow decay rate (*t*_1/2_ ~ 11-d) [9], it can be used to calculate myofibrillar FSR (myoFSR) in vivo in humans over longer-time intervals (> 24 h), under ‘free-living’ conditions [9,10,11,12]. Continuous ‘integrative’ day-to-day myoFSR measurement incorporates several concomitant variables that do not factor in short-duration lab-based studies (e.g., dietary intake, distribution, feeding pattern, physical (in)activity, sleep-pattern, hormonal/diurnal variation). As such, deuterated labelling methods can be used to provide important information on the role that exercise and/or nutrition has on the regulation of myoPS in free-living humans. 

From an athlete’s perspective, dietary/nutritional interventions that augment recovery from, and adaptation to, RT are desirable. There is strong evidence to suggests that high-quality supplemental protein plays an important role in optimising recovery and the reconditioning of skeletal muscle after exercise [13,14,15,16]. However, the putative beneficial effects of protein supplementation on myoPS have not been tested in resistance-trained individuals over longer periods of training (i.e., >24 h) with multiple bouts of RT. Therefore, the aim of this study was to concurrently assess the impact of protein supplementation on myoPS and muscle recovery indices over the course of an intensified period of RT. 

In this study, we recruited a cohort of young healthy resistance-trained men and applied the D_2_O-method to measure myoPS over a 7-d RT protocol, whilst also monitoring indices of muscle recovery daily. Over the course of the study, participants completed three lower-body RT-sessions on non-consecutive days. The RT protocol was combined with morning ingestion (~0.5 h pre-exercise on RT-days) of whey protein (0.33 g⸱kg^−1^·d^−1^) or an isonitrogenous control. We hypothesised that compared to the control, dietary supplementation of whey protein would enhance both myoPS and recovery over the 7-d RT-protocol.

## 2. Materials and Methods 

### 2.1. Ethical Statement and Study Design

Participants were informed of the risks and benefits before providing written informed consent. Ethical approval was granted by the University of Limerick Education and Health Sciences Research Ethics Committee (2016_12_09 EHS), conforming to standards set by the Declaration of Helsinki and pre-registered at clinicaltrials.gov with the study identifier NCT03297151. In a double-blind parallel-group design, participants were block-randomised (*n* = 8 per group) to either the whey protein supplement group (WHEY) or the control supplement group (CON). 

### 2.2. Participants

Eligibility criteria were: (i) men aged 18 to 35 years; (ii) six-months of RT-experience (>3 h∙wk^−1^); (iii) able to competently perform a 1.25 kg∙kg^−1^ barbell back-squat 1RM; (iv) no current injury, illness, medication or history of chronic disease; (v) lactose tolerant. To verify eligibility, back-squat strength/competency tests, medical history and RT-experience were assessed the week prior to study commencement. Sixteen participants in total were recruited and, following informed consent, proceeded to the study. One participant (WHEY) failed to comply with the study protocol and was excluded from the final analysis.

### 2.3. Pre-study Dietary Assessment, Control and Supplementation

Before starting the study, participants completed a 7-d record of their habitual dietary intake, feeding pattern, exercise training and activities of daily living (ADL). Dietary records were analysed on Nutritics® software and a standardised 7-d meal plan was constructed, prepared, and monitored by a registered dietician and resident chef. The energy and nutrient content of the meal plan was based on participants’ average habitual intake (Appendix A), being standardised and scaled to body mass per diem for each participant (Appendix B). Every test-day, participants were provided with three main meals and two snacks, to be consumed at 3 h intervals during waking hours. Protein content of the study diet met the current recommendations of the American College of Sports Medicine (ACSM) (i.e., 1.2 to 2.0 g·kg^−1^·d^−1^) [17], International Olympic Committee (IOC) (i.e., 1.6 to 2.2 g·kg^−1^·d^−1^) [18] and micronutrient daily recommended intakes [19]. 

In order to maximise postexercise/postprandial myoPS and ensure compliance to the study diet, morning ingestion of 0.33 g·kg^−1^·d^−1^ of protein, from a whey protein concentrate supplement [20], after an overnight fast (~10 h postabsorptive, ~0.5 pre-exercise [21]), was conducted. The control group consumed an isonitrogenous formulation of nonessential amino acids that does not stimulate myoPS (Carbery Food Ingredients, Ireland) (Appendix C) [7]. Supplements were mixed with 500 ml water, identically flavoured (vanilla) and administered in a double-blind fashion. The first meal of the day was consumed 3 h following supplement ingestion. 

### 2.4. Study Protocol

**Figure 1:**
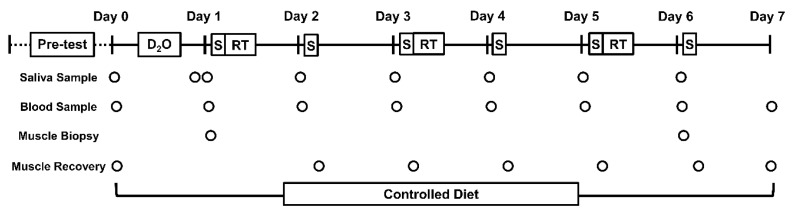
Study overview. D_2_O, deuterium oxide bolus (400 ml 70 atom %); RT, lower-body resistance-training session; S, supplement ingestion (0.33 g·kg·d^−1^ whey protein or isonitrogenous control) tested in a randomised double-blind parallel-group design. Pre-test measures included physical/medical screen, familiarisation, body composition and dietary assessment. Biopsies were sampled from *m. vastus lateralis.* Muscle recovery was assessed via peak isometric squat force, countermovement jump height, lower-body muscle soreness and serum creatine kinase.

Figure 1 depicts the study protocol. Participants reported to the lab each morning after an overnight fast for eight consecutive days. Day 0 body mass and stature were taken via a scale (Tanita MC, 180-MA, UK) and stadiometer (Seca, Birmingham, UK). Body composition was measured by a dual-energy x-ray absorptiometry (DXA, Lunar iDXA™, GE Healthcare) scan. Following this, participants completed baseline muscle function tests. After testing participants ingested 400 ml of D_2_O over a 4 h period (70 atom %, 100 mL·h^−1^), provided blood and saliva samples pre-ingestion and +2 h post-ingestion to assess deuterium enrichment. Each day thereafter, participants present to the laboratory and provided a fasted/resting blood and saliva sample, ingested the supplement then completed muscle function tests. On days 1, 3 and 5, a RT session was undertaken after muscle function tests (~ 0.5 h post-ingestion). Percutaneous microneedle biopsy samples of *m. vastus lateralis* were collected on day 1 and day 6 (between blood/saliva and muscle function tests), as reported previously [22]. Following completion of data collection, participants were free to undertake ADL, but were asked to refrain from any formal exercise and consume only food provided to them by the research team. Compliance was confirmed by daily meal-container inspection and self-report questionnaire on each return visit to the lab. 

### 2.5. Resistance Training Protocol

Participants performed sets of 10 barbell back-squats to repetition failure. For the back-squat, participants fixed a loaded barbell (70% 1RM) across their shoulders on the trapezius (above the posterior aspect of the deltoids), flexed hips and knees until thighs were parallel to the floor, then extended hips and knees to a standing position [23]. Each repetition was performed at a cadence of 6 s (controlled by a metronome or visible timer). Three min rest was given between sets (25% duty cycle). Repetition failure was operationally defined as the inability to complete a repetition, or an observable change in technical execution increasing injury risk (e.g., spinal flexion, valgus collapse, asymmetry, and imbalance) [23]. The number of repetitions was recorded at failure. To ensure compliance and best practice, the RT sessions were supervised by at least two experienced strength and conditioning professionals.

### 2.6. Muscle Recovery Parameters

#### 2.6.1. Isometric Squat Force

Measurement of maximal voluntary isometric squat (ISq) force was taken on a custom-made squat rack with a fixed barbell, adjustable in height, positioned above two force plates (AMTI, Watertown, MA, USA) bolted to the floor. Squat position was fixed (110° knee angle) and foot positioning on the force-plates was marked, recorded at baseline and repeated for each subsequent measurement. Once positioned, in contact with the barbell, force was tared, and on an audible cue, standard verbal encouragement was given to push into the barbell as fast and forcefully as possible for at least 3-s. This was repeated three times with 3 min rest between attempts. Ground reaction force data was sampled at 1 kHz and excluded if any countermovement was noted. Peak isometric force was defined as the highest 1-s average ground reaction force attained during the three attempts. The coefficient of variation (CV) at baseline was 7.6%. 

#### 2.6.2. Countermovement Jump Height

A countermovement jump (CMJ) test was used to assess lower-body dynamic strength. CMJ height was calculated from flight-time (obtained from ground reaction force data (AMTI, Watertown MA, USA)). To prevent non-vertical movement between take-off and landing, participants were instructed to place hands on hips and keep their body straight throughout the jump, landing in the same upright toe-first position as for take-off. Participants performed three CMJs with 3 min rest between attempts. Peak jump height (calculated from flight time [24]) was the highest jump of the three attempts. Measurement CV at baseline was 5.4 %. Participants were familiarised with the ISq and CMJ tests the week prior to starting the study.

#### 2.6.3. Serum Creatine Kinase

Creatine kinase (CK) activity was measured as an indirect marker of muscle damage. For analysis, venous blood samples were allowed to clot at room temperature before centrifugation at 1750 *g* for 10 min at 22°C. Serum was then aliquoted and stored at −80°C. CK was quantified via spectrophotometry using an enzymatically coupled assay (Sigma-Aldrich, St. Louis, MO, USA). Derived from two-level quintuplet quality-controls, the CV was calculated as 5% within and 12% between each assay. 

#### 2.6.4. Muscle Soreness

Immediately following the ISq tests, participants were asked to quantify general subjective feelings of lower-body muscle soreness during the ISq, using a 10 cm visual analogue scale (VAS). VAS descriptors were ‘no pain’ (0 cm), to ‘worst possible pain’ (10 cm). 

### 2.7. Muscle Analysis

#### 2.7.1. Myofibrillar Protein Synthesis

To measure body water deuterium enrichment, pure body water was extracted through heating 100 µL of the saliva sample, before being condensed and transferred to an autosampler vial ready for injection into a high-temperature conversion elemental analyser (TC-EA) (Thermo Finnigan, Thermo Scientific, Hemel Hempstead, UK) connected to an isotope ratio mass spectrometer (IRMS) (Delta V Advantage, Thermo Scientific). To minimise carryover between samples, each sample was injected four times, with the average of the last three injections used for analysis. For accuracy, a known standard D_2_O enrichment curve was run alongside samples. 

To measure myofibrillar protein alanine enrichment, 30 mg of muscle was homogenised in ice-cold homogenisation buffer, vortexed for 10 min and centrifuged at 13,000 *g* for 10 min at 4 °C before the supernatant was removed. The pellet was solubilized in 0.3 M NaOH before centrifugation at 13,000 *g* for 10 min at 4 °C to separate the insoluble collagen fraction. The myofibrillar containing supernatant was subsequently collected and the proteins were precipitated by the addition of 1 M perchloric acid (PCA). For the plasma proteins, 100 µL of the sample was precipitated using 100 µL ice-cold ethanol then separated through centrifugation. Protein-bound amino acids were hydrolysed overnight in 0.1 M HCl and Dowex H^+^ resin at 110 °C, before elution with 2 M NH_4_OH and then evaporated to dryness. Amino acids were derivatised to their n-methoxycarbonyl methyl esters by resuspension in 60 µL distilled water and 32 µL methanol, before vortexing and the addition of 10 µL pyridine and 8 µL methylchloroformate. Then, 100 µL of 0.001 M NaHCO_3_ was added, before samples were vortexed and extracted in 100 µL of chloroform, addition of a molecular sieve to used remove any remaining water. Samples were transferred into a new small volume chromatography vial insert. The deuterium enrichment of protein-bound alanine was measured by sample injection and assessment by gas chromatography–pyrolysis–isotope ratio mass spectrometry (GC-Pyr-IRMS, Delta V Advantage, Thermo Scientific). Samples were injected in triplicate, alongside a standard curve of known L-alanine-2,3,3,3-d4 enrichment. 

Integrated myoFSR was calculated from the incorporation of deuterium-labelled alanine into the myofibrillar protein fraction, using body water as a surrogate for precursor enrichment (corrected for the mean number of deuterium moieties incorporated per alanine (3.7) and the 11 hydrogen atoms within the alanine derivative) [9,12]. The equation was:

$$\mathrm{myoFSR}=-\ln\left(\frac{1-\left[\frac{{\mathrm{APE}}_{\mathrm{Ala}}}{{\mathrm{APE}}_{p}}\right]}{t}\right)$$

where APE_ala_ is deuterium enrichment of protein-bound alanine over the course of the study; APE_p_ is mean precursor enrichment over the study, and t is the time between biopsies (day 1 and day 6) [9,10]. 

#### 2.7.2. Muscle RNA

Fifteen milligrams of muscle was freeze-dried before homogenisation in 0.2 M of PCA, then centrifugation at 11,680 *g* and washed in 0.2 M PCA. The pellet was then suspended in 0.3 M NaOH at 37 °C for 30 min. Proteins were precipitated with 1 M PCA before centrifugation at 9000 *g.* RNA containing supernatant was collected then measured by spectrophotometry (NanoDrop Lite, Thermo Scientific). 

### 2.8. Statistical Analysis

Descriptive statistics were mean (SD). Group and time difference were reported as mean [95 % CI]. For statistical analysis normality and homogeneity were confirmed prior to performing parametric statistical tests. Mixed-model ANOVA was used to verify group × time interaction (i.e., supplement-effect). Independent t-tests were used to assess differences between groups. Repeated-measures ANOVA and paired t-tests were used to assess time effects on pooled data (i.e., RT-effect). The critical significance level was set as α = 0.05. A false-discovery rate correction was used to adjust for familywise error [25]. The magnitude of the change was examined by effect size (ES) (Cohen’s d [26]). A sample size estimate was derived from previously published data (1 – β = 0.8, α = 0.05, *d_z_* > 1.0) [5,9]. Statistical analysis was performed in SPSS version 25 (IBM, Chicago, IL, USA) and Microsoft Excel™.

## 3. Results

### 3.1. Participants and Dietary Compliance

Baseline characteristics are presented in Table 1. There were no differences between groups for: age, stature, body mass, lean body mass (LBM), LBM index (LBMI), body fat %, back-squat 1RM or RT-experience (*p* > 0.276). Participants were weight-stable over the study protocol (± 2 kg). Meal and snack containers were inspected daily to verify adherence to the study diet. Participants consumed 98% of their required total dietary protein intake. 

### 3.2. Myofibrillar Protein Synthesis

**Figure 2:**
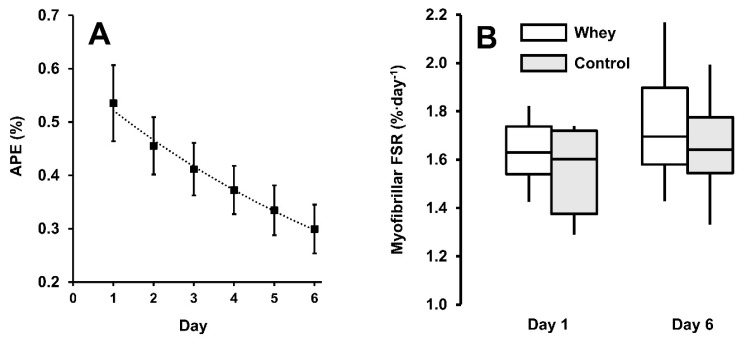
Body water deuterium enrichment (atom percent excess (APE) (%)) **(A)**, data are mean ± SD. Myofibrillar fractional synthetic rate (FSR) **(B)**, boxplot data are median (horizontal line), interquartile range (box), minimum and maximum (whiskers), there was no difference between whey (white) and control (grey) groups (*p* = 0.771).

Twenty-four hours after D_2_O bolus ingestion, body water enrichment increased to 0.54% (0.08%), then followed a single-phase exponential decay curve (r^2^ = 0.993) to 0.30% (0.05%) (Figure 2A). No difference in myoFSR was observed between groups at baseline (WHEY = 1.62 (0.13) %∙d^−1^ vs. CON = 1.59 (0.19) %∙d^−1^, *p* = 0.776). Whey protein supplementation did not increase myoFSR (0.01 [−0.10, 0.11] %∙d^−1^, *p* = 0.771, ES < 0.1) (Figure 2B). Over time, a medium ES was calculated for RT, increasing myoFSR, but failed to reach the critical significance level (1.61 (0.13) %∙d^−1^ to 1.72 (0.24) %∙d^−1^, 0.11 [−0.01, 0.22] %∙d^−1^, *p* = 0.106, ES = 0.5, *n* = 15).

### 3.3. Muscle Recovery Measures and Resistance Training Performance

**Figure 3:**
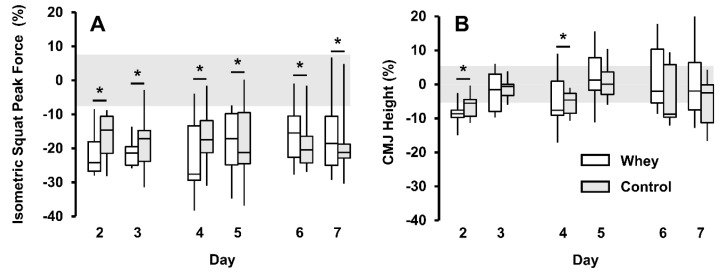
Isometric squat peak force % change **(A)**. Countermovement jump (CMJ) height % change **(B)**. Boxplot data are median (horizontal line), interquartile range (box), minimum and maximum (whiskers). Grey band represents measurement uncertainty (7.6% **(A)** and 5.4% **(B)**). *Different from baseline (*p <* 0.05). There was no difference between whey (white) and control (grey) groups for either measure (*p >* 0.441).

Participants completed 8.9 (1.9) sets per session. A deficit in ISq force was observed at all time-points after the first RT session (day 2 to day 7) (*p* < 0.001, ES > 1.8) (Figure 3A) and a decrease in CMJ height was observed +24 h after session 1 (day 2) (−8% [−5%, −10%], *p* < 0.001, ES = 1.3) and +24 h after session 2 (day 4) (−5% [−1%, −9%], *p* = 0.011, ES = 0.7) (Figure 3, Appendix D). Whey protein supplementation did not improve the number of reps to failure during RT (P = 0.441) or ISq force (*p* = 0.923) or CMJ height (*p* = 0.441) recovery between RT-bouts. 

An increase in serum CK was observed +24 h after session 1 (day 2) (138 (87) to 215 (89) IU∙L^−1^, 94% [59%, 130%], *p* < 0.001, ES = 1.3) then returned to baseline thereafter (Appendix D). Whey protein supplementation did not affect serum CK (*p* = 0.989). An increase in lower-body muscle soreness was reported at all time-points after session 1 (day 2 to day 7) (*p* < 0.009, ES > 0.9) (Appendix D); however, whey protein supplementation did not reduce soreness (*p* = 0.390)

### 3.4. Muscle RNA

Muscle RNA at baseline was 2.5 μg·mg^−1^ (WHEY = 2.7 (0.4) μg·mg^−1^, CON = 2.3 (0.1) μg·mg^−1^). There was no supplement (*p* = 0.856) or RT-effect (*p* = 0.848, ES < 0.1).

## 4. Discussion

In the present study, we investigated the effect of whey protein supplementation on myoPS and indices of muscle recovery, over a 7-d intensified RT protocol. Our data show that in a cohort of young healthy resistance-trained men, daily supplementation of whey protein did not augment myoFSR or any measure of muscle recovery over multiple days of RT.

In a recent study, using the D_2_O-method, we reported that acute ingestion of 0.33 g·kg^−1^ of whey protein increased postexercise myoFSR, ~30 % for ~5 h, in a young healthy resistance-trained cohort [7]. In the present study, the D_2_O-tracer technique was employed to measure myoFSR, but this time measuring the effect of 0.33 g·kg^−1^·d^−1^ of supplemental whey protein over multiple days of RT. We believe the conflicting results between this study and the acute lab-based studies [4,5,6,7] are due to the incorporation of several ‘free-living’ variables over the 7-d assessment (e.g., physical (in)activity, periods of fasting between meals and during sleep, hormonal and diurnal variation), which confounded or ‘diluted’ the acute feeding effect following supplement ingestion [7]. 

In response to RT, there is evidence to suggest that day-to-day, D_2_O-derived, measures of myoPS are predictive of longer-term physical changes to the muscle [10,11], whereas acute lab-based measures of myoPS are not [8]. To our knowledge, this is the first study examining the impact of protein supplementation on myoPS over multiple days of RT in resistance-trained individuals. To maintain ecological validity, dietary standardisation (i.e., intake and feeding pattern) in the present study was based upon participants’ habitual intake, recorded prior to starting the study (Appendix A and Appendix B). Consequently, the protein supplement intervention only constituted 17% (i.e., 0.33 g·kg^−1^⸱d^−1^ of 2.0 g·kg^−1^⸱d^−1^) of the total daily protein intake. We believe that of the ‘free-living’ factors, it was the participants’ relatively high dietary protein intake (i.e., 1.7 g·kg^−1^⸱d^−1^) [17,18] that likely diminished any additive effect that the supplemental protein may have had on myoPS. 

A number of acute lab-based studies have shown a dose-dependent relationship between protein ingestion and postprandial/postexercise myoFSR, plateauing at > 0.31 g·kg^−1^ (~ 20 g of protein) [4,5,20]. However, the dose-response per diem, has not been as well characterised with current recommendations for athletes ranging from 1.2 to 2.2 g·kg^−1^⸱d^−1^ [17,18]. It is well known that myoPS is a key driver of skeletal muscle mass gain during RT. Results from a biphasic regression (meta)analysis, across several studies, suggest that supplementation beyond a protein intake of 1.6 g·kg^−1^⸱d^−1^ does not enhance RT-induced increases in fat-free mass [27]. Indeed, findings from the present study supports this notion, showing that appropriately timed (postabsorptive, temporal proximity to RT) and dosed (0.33 g·kg^−1^) high-quality protein supplementation, increasing total daily protein intake from 1.7 g·kg^−1^⸱d^−1^ from 2.0 g·kg^−1^⸱d^−1^, confers no further increase in myoPS. 

Pooled data analysis revealed that, independent of supplements group, the RT-protocol failed to evoke a substantial increase in myoPS, not reaching the critical significance level (*n =* 15, *P* = 0.106, ES = 0.5). However, several studies, with similar sample sizes, have reported statistically significant increases in D_2_O-derived measures of myoPS in response to RT [10,11,12]. Prior to starting the study, we confirmed that participants were regularly partaking in RT and consuming adequate levels of dietary protein. As such, the minimal increase in myoPS may be suggestive of a ‘muscle-full’ effect, whereby the muscle had already reached, or was close to, its FSR set-point of accommodation prior to starting the study [3]. To examine these null findings further, we analysed muscle RNA data post-hoc. As ~80% of the total cellular RNA is ribosomal (rRNA), a rapid increase in RNA is indicative of rRNA synthesis [28], which is associated with increases in translational efficiency, capacity and myoPS response to anabolic stimuli [10,29,30,31]. Indeed, a cross-study analysis revealed that an untrained age-matched cohort had significantly lower RNA content, myoFSR and muscle protein content compared to our resistance-trained cohort [10]. Therefore, we speculate that rapid expansion of the ribosomal pool had occurred prior to starting the study in our participants, limiting room to further stimulate myoFSR over the RT-intervention.

In addition to the muscle metabolic response, from a practical standpoint we were also interested in examining the effect of supplemental whey protein on postexercise muscle recovery during the RT-protocol. Indeed, several studies have reported that protein supplementation facilitates temporal recovery after RT by improving neuromuscular function [15,16], exercise performance [13,15], reducing muscle damage [13] and muscle soreness [32], providing an obvious benefit to athletes who regularly engage in RT. Although alternative mechanisms have been proposed [32], it is commonly accepted that supplemental dietary protein enhances muscle recovery by accelerating the reconditioning, specifically of the myofibrillar (force-generating) protein fraction. To our knowledge, this is the first study to concurrently assess myoPS alongside indices of recovery. In line with our metabolic data, whey protein supplementation appeared to have no beneficial effect on any muscle recovery parameter (i.e., ISq or CMJ force output, RT-performance, serum CK or muscle soreness), at any time-point over the course of the RT-protocol. 

Despite the null findings, this study highlights the important role that some, frequently overlooked, ‘free-living’ variables may have on the regulation of muscle metabolism and recovery in response to RT. In particular, we highlight the influence that participants’ habitual dietary intake and/or feeding pattern, may have on dietary supplement interventions. As such, we note that it is probable that some of the inherent baseline characteristics of our study population (i.e., adequate dietary protein intake and previous RT-experience) affected the results, thus, limiting our findings to young healthy resistance-trained men. Further research is still warranted to confirm if protein supplementation, with or without exercise, enhances day-to-day measures of myoPS in other relevant populations (e.g., sarcopenic, injured/immobilised, sedentary/untrained individuals and/or those with restricted/low-dietary protein intake). Nevertheless, this study provides important information and insight into how the D_2_O methodology can be applied in a free-living setting.

## 5. Conclusions

In conclusion, 0.33 g·kg^−1^⸱d^−1^ of whey protein supplementation over an intensified 7-d RT protocol fails to enhance integrative rates of myoPS or recovery in young health resistance-trained men consuming adequate amounts of dietary protein (1.7 g·kg^−1^⸱d^−1^).

## Figures and Tables

**Table 1:** Participant Baseline Characteristics.

	WHEY (*n* = 7)	CON (*n* = 8)	*p*-value
Age (y)	24 (4)	23 (5)	0.561
Stature (m)	1.81 (0.08)	1.77 (0.04)	0.276
Body Mass (kg)	81 (10)	77 (17)	0.649
LBM (kg)	63 (8)	60 (10)	0.488
LBMI (kg·m^−2^)	19 (2)	19 (3)	0.830
Body fat (%)	17 (6)	18 (6)	0.807
1RM (kg·kg^−1^)	1.5 (0.3)	1.5 (0.3)	0.852
RT-experience (y)	2.4 (1.1)	2.6 (1.5)	0.783

Data are mean (SD). LBM, lean body mass; LBMI, lean body mass index 1RM, one repetition maximum back-squat per kg body mass; RT, resistance training; WHEY, whey protein concentrate group; CON, control group.

## Appendix A

**Table A1:** Habitual Diet.

Energy		Protein		Frequency	Timing
kJ·kg^−1^⸱d^−1^	kJ·kg^−1^⸱EO^−1^	g·kg^−1^⸱d^−1^	g·kg^−1^⸱EO^−1^	EO·d^−1^	h·EO^−1^.
154 (26)	31 (6)	1.9 (0.5)	0.39 (0.1)	5 (1)	3.3 (0.6)

Participants habitual dietary intake and feeding pattern. Data are mean (SD). Dietary intake is scaled to body mass (kg). EO, eating occasion (i.e., meal or snack). Timing (h**·**EO^−1^) represents time between EOs during waking hours.

## Appendix B

**Table A2:** Study Diet.

	Energy (kJ·kg^−1^)	Protein (g·kg^−1^)	CHO (g·kg^−1^)	Fat (g·kg^−1^)	Frequency (d^−1^)
Snacks	16	0.34	0.43	0.07	2
Meals	38	0.34	1.22	0.29	3
Daily Intake from Food	146	1.7	4.5	1.0	5

Standardised study diet scaled to body mass (kg). Eating occasions consisted of snacks and meals consumed at 3 h intervals during waking hours. CHO, carbohydrate.

## Appendix C

**Table A3:** Supplement Amino Acid Composition.

	WHEY			CON		
	mg·kg^−1^	%	Dose (g)	mg·kg^−1^	%	Dose (g)
Alanine	17	5	1.4 (0.2)	33	10	2.6 (0.6)
Arginine	8	2	0.7 (0.1)	0	0	0
Aspartic acid	35	11	3.1 (0.4)	41	12	3.2 (0.7)
Cysteine	8	2	0.6 (0.1)	0	0	0
Glutamic acid	56	17	4.9 (0.6)	120	36	9.3 (2.0)
Glycine	6	2	0.5 (0.1)	13	4	1.0 (0.2)
Histidine	6	2	0.5 (0.1)	0	0	0
Isoleucine	20	6	1.8 (0.2)	0	0	0
Leucine	34	10	3.0 (1.4)	0	0	0
Lysine	30	9	2.7 (0.3)	0	0	0
Methionine	7	2	0.6 (0.1)	0	0	0
Phenylalanine	10	3	0.9 (0.1)	0	0	0
Proline	19	6	1.7 (0.2)	53	16	4.2 (0.9)
Serine	17	5	1.4 (0.2)	45	14	3.5 (0.8)
Threonine	23	7	2.1 (0.3)	0	0	0
Tryptophan	7	2	0.6 (0.1)	0	0	0
Tyrosine	9	3	0.8 (0.1)	25	8	2.0 (0.4)
Valine	19	6	1.7 (0.2)	0	0	0
EAA	160	48	13.7 (1.7)	0	0	0
NEAA	170	52	15.2 (1.9)	330	100	25.8 (5.6)
TAA	330	100	28.9 (3.6)	330	100	25.8 (5.6)

Amino acid composition of the whey protein concentrate (WHEY) and nonessential amino acid control (CON). Data are mg∙kg^−1^ body mass, % total mass and absolute gram dose (mean (SD)). Supplement dose was isonitrogenous and scaled to body mass (0.33 g·kg^−1^). EAA, essential amino acids; NEAA, nonessential amino acids; TAA, total amino acid content.

## Appendix D

**Table A4:** Recovery Indices.

	Group	Baseline	Day 2	Day 3	Day 4	Day 5	Day 6	Day 7
ISq Force (*N*)	WHEY	827 (195)	670 (146)	648 (149)	643 (192)	677 (194)	672 (169)	709 (173)
	CON	740 (235)	609 (172)	593 (162)	608 (191)	599 (212)	591 (189)	582 (148)
	Pooled	776 (205)	626 (154)*	610 (154) *	611 (172) *	628 (183) *	615 (163) *	632 (162) *
CMJ Height (cm)	WHEY	38 (5)	35 (4)	37 (5)	36 (4)	38 (5)	32 (14)	39 (5)
	CON	37 (6)	33 (6)	35 (7)	33 (6)	35 (6)	29 (13)	33 (5)
	Pooled	37 (6)	34 (5) *	36 (6)	35 (5) *	37 (6)	32 (10)	36 (6)
VAS (cm)	WHEY	0.4 (0.3)	2.8 (1.4)	3.0 (1.8)	2.4 (1.4)	1.9 (1.5)	1.9 (1.8)	1.8 (2.2)
	CON	0.3 (0.3)	1.7 (1.1)	1.5 (2.1)	1.3 (1.7)	1.5 (1.6)	2.8 (2.0)	1.5 (2.2)
	Pooled	0.4 (0.3)	2.3 (1.3)*	2.3 (2.1) *	1.8 (1.6) *	1.7 (1.5) *	2.4 (1.9) *	1.6 (2.1) *
CK (IU∙L^−1^)	WHEY	157 (87)	230 (88)	166 (61)	188 (78)	153 (67)	182 (82)	158 (79)
	CON	130 (38)	208 (42)	176 (39)	181 (36)	157 (30)	174 (54)	152 (53)
	Pooled	138 (87)	215 (89) *	168 (68)	181 (78)	153 (66)	172 (93)	149 (90)

Data are mean (SD). WHEY, whey protein group; CON, control group; ISq force, isometric squat peak force; CMJ, countermovement jump; VAS, visual analog scale (muscle soreness); CK, creatine kinase. * Significantly different to baseline (*p* < 0.05).
